# Luteolin alleviates post‐infarction cardiac dysfunction by up‐regulating autophagy through Mst1 inhibition

**DOI:** 10.1111/jcmm.12714

**Published:** 2015-11-05

**Authors:** Jianqiang Hu, Wanrong Man, Min Shen, Mingming Zhang, Jie Lin, Tingting Wang, Yu Duan, Congye Li, Rongqing Zhang, Erhe Gao, Haichang Wang, Dongdong Sun

**Affiliations:** ^1^ Department of CardiologyXijing HospitalFourth Military Medical UniversityXi'anChina; ^2^ Center for Translational MedicineTemple University School of MedicinePhiladelphiaPAUSA

**Keywords:** luteolin, myocardial infarction, autophagy, mammalian Ste20‐like kinase 1

## Abstract

Myocardial infarction (MI), which is characterized by chamber dilation and LV dysfunction, is associated with substantially higher mortality. We investigated the effects and underlying mechanisms of Luteolin on post‐infarction cardiac dysfunction. Myocardial infarction was constructed by left anterior descending coronary artery ligation. *In vitro*, cultured neonatal cardiomyocytes subjected to simulated MI were used to probe mechanism. Luteolin significantly improved cardiac function, decreased cardiac enzyme and inflammatory cytokines release after MI. Enhanced autophagic flux as indicated by more autophagosomes puncta, less accumulation of aggresomes and P62 in the neonatal cardiomyocytes after hypoxia was observed in the Luteolin pre‐treatment group. Western blot analysis also demonstrated that Luteolin up‐regulated autophagy in the cardiomyocytes subjected to simulated MI injury. Furthermore, Luteolin increased mitochondrial membrane potential, adenosine triphosphate content, citrate synthase activity and complexes I/II/III/IV/V activities in the cardiomyocytes subjected to simulated MI injury. Interestingly, mammalian sterile 20‐like kinase 1 (Mst1) knockout abolished the protective effects of Luteolin administration. Luteolin enhances cardiac function, reduces cardiac enzyme and inflammatory markers release after MI. The protective effects of Luteolin are associated with up‐regulation of autophagy and improvement of mitochondrial biogenesis through Mst1 inhibition.

## Introduction

Myocardial infarction remains the leading cause of morbidity and mortality worldwide 1, 2. Percutaneous coronary intervention and intensive pharmacotherapy can normalize coronary perfusion, reduce adverse ventricular remodelling and thus improve myocardial salvage after MI 3. However, a large variety of patients develop LV remodelling and progressive heart failure after MI. The mortality after MI has worsened in recent years 4, 5, 6, 7. The restoration of cardiac function in such situations remains a major challenge 8.

Luteolin is a polyphenolic compound which is widely distributed in vegetables, fruits and medicinal herbs 9, 10, 11. Preclinical studies have shown that Luteolin possesses a variety of biological and pharmacological activities. Moreover, Luteolin has been reported to reduce the mortality from coronary artery diseases and serves as a potential candidate for the prevention and treatment of cardiovascular diseases 12.

We previously demonstrated that Luteolin preserved cardiac function, reduced infarct size and cardiomyocyte apoptotic rate after ischaemia/reperfusion (I/R) injury in diabetic rats 13. In addition, Luteolin has been shown to improve contractile function and attenuates apoptosis following I/R injury in adult rat cardiomyocytes 14. However, the underlying mechanism by which it exerts cardioprotection against MI has not been fully elucidated.

Autophagy is a physiological process in which a cell digests its own constituents *via* the lysosomal degradative pathway. Basal levels of autophagy are important for maintaining cellular homoeostasis and for protecting cells against excess or dysfunctional organelles 15. In the heart, inefficient autophagy or its absence leads to poor myocardial performance. Furthermore, enhancing autophagy can also promote cell survival in response to stress including heart failure 16 and ischaemic cardiomyopathy 17.

Mammalian sterile 20‐like kinase 1 is a mammalian homologue of *Drosophila* Hippo, the master regulator of cell death, proliferation and organ size in flies. It is the chief component of the mammalian Hippo pathway and promotes apoptosis and inhibits compensatory cardiac hypertrophy, playing a critical role in mediating heart failure. Mst1 has been reported to regulate autophagy, apoptosis, proliferation and organ size 18, 19. Overexpression of Mst1 promoted cardiac myocyte apoptosis and exacerbated adverse remodelling in response to I/R injury 18, whereas inhibition of endogenous Mst1 reduces the size of MI and prevents cardiomyopathy 18, 19. As Luteolin has been proven to modulate autophagy in many circumstances 20, we therefore attempted to elucidate whether cardiomyocyte autophagy was involved in mediating the protective effects of Luteolin against MI.

## Materials and methods

### Animals and treatment

All animal protocols were approved by the Fourth Military Medical University Ethic Committee on Animal Care (Approval ID: 2013067) and all experiments were performed in adherence with the National Institutes of Health Guidelines on the Use of Laboratory Animals. Eight‐ to 12‐ week‐old C57BL/6 wild‐type mice were purchased from Jackson Laboratories (Bar Harbor, MI, USA) and randomly allocated into the following groups with *n* = 30 each: (*i*) sham group (Sham); (*ii*) MI group; (*iii*) MI +DMSO group and (*iv*) MI +Luteolin.

Mst1 transgenic (Tg) and Mst1 knockout (Mst1^−/−^) mice were established at K&D Gene Technology (WuHan, China) (C57BL/6 background). Western blot analysis and real‐time PCR (RT‐PCR) were used to screen Mst1Tg and Mst1^−/−^ mice. Eight‐ to 12‐ week‐old Mst1Tg and Mst1^−/−^ were randomly allocated into the following groups with *n* = 30 each: (*i*) Mst1Tg+sham group (Mst1Tg+Sham); (*ii*) Mst1Tg+MI group; (*iii*) Mst1Tg+ MI+Luteolin group; (*iv*) Mst1^−/−^+sham group (Mst1^−/−^Sham); (*v*) Mst1^−/−^+ MI group and (*vi*) Mst1^−/−^ + MI +Luteolin group.

Before constructing the MI model, Luteolin (10 μg/kg) (99%; Sigma‐Aldrich, St Louis, MO, USA) was dissolved in DMSO and was injected intraperitoneally for 3 days.

### Myocardial infarction surgery

As described previously 21, the MI animal model was constructed by the left anterior descending coronary artery ligation. Sham‐operated control animals underwent a similar surgery without ligation of the artery. The mice were allowed to recover in a cage with the temperature maintained at 37°C under human care.

### Adenovirus constructs

Adenovirus encoding GFP‐LC3 was purchased from GeneChem Technology Ltd (Shanghai, China). Adenoviruses expressing a short hairpin (sh) RNA directed against Mst1, harbouring Mst1, control vectors (Ad‐LacZ, Ad‐sh‐LacZ) and GFP‐mRFP‐LC3 were purchased from Hanbio Technology Ltd (Shanghai, China). The titres of adenoviruses were 1 × 10^10^ PFU/ml. The MOI of adenovirus was 80:1. The shRNA sequence targeting mouse Mst1 is GCCCTCACGTA GTCAAGTATT.

### Neonatal cardiomyocyte culture

Primary cultures of cardiomyocytes were harvested from the ventricle of neonatal C57BL/6 mice (1‐day‐old). The neonatal mice were sterilized with 75% ethanol and the hearts were removed rapidly and rinsed in the cold PBS to clean out the remaining blood. The Myocardium specimen were then cut into pieces and digested by collagenase type 2 (Sigma‐Aldrich) until the tissue blocks had disappeared. The suspension was collected and blended in with the completed medium to stop digestion. After that, the mixture liquid was centrifuged (800 × g for 5 min.) and then the supernatant was removed. The cardiomyocyte‐rich fraction was resuspended in complete medium containing 20% foetal bovine serum (Gibico, California, USA). Differential adhesion was used to separating the cardiomyocytes from fibroblasts effectively. The dissociated cells were plated in a culture flask at 37°C for 1 hr to ensure that the fibroblasts could load on the bottom of the culture flask, whereas the cardiomyocytes were still suspended in the medium. Then the cardiomyocytes were replated on confocal dishes. These procedures were carried out at 37°C in the presence of 95% O_2_ and 5% CO_2_. After 48 hrs, the adenovirus harbouring GFP‐LC3 was transduced and the adenoviruses harbouring Mst1 (Ad‐Mst1), Mst1 shRNA (Ad‐sh‐Mst1) were transduced 24 hrs after transduction of GFP‐LC3. After 4 hrs, the cardiomyocytes were administrated either in the absence or presence of Luteolin for 48 hrs as described previously 22, and then treated with hypoxia for 8 hrs. Cardiomyocytes were randomly allocated into the following groups: (*i*) Control (CON); (*ii*) Hypoxia (H); (*iii*) Hypoxia+DMSO (H+DMSO); (*iv*) Hypoxia+Luteolin (H+Luteolin); (*v*) Control (CON); (*vi*) Ad‐LacZ+Hypoxia (Ad‐LacZ+H); (*vii*) Ad‐Mst1+Hypoxia (Ad‐Mst1+H); (*viii*) Ad‐LacZ+Hyoxia+Luteolin (Ad‐LacZ+H+Luteolin); (*ix*) Ad‐Mst1+ Hypoxia+ Luteolin (Ad‐Mst1+H+Luteolin); (*x*) Ad‐sh‐LacZ+Hypoxia (Ad‐sh‐LacZ+H); (*xi*) Ad‐sh‐Mst1+ Hypoxia (Ad‐sh‐Mst1+H); (*xii*) Ad‐sh‐LacZ+Hypoxia+Luteolin (Ad‐sh‐LacZ+ H+ Luteolin) and (*xiii*) Ad‐sh‐Mst1+Hypoxia+Luteolin (Ad‐sh‐Mst1+H+Luteolin).

### Detection of GFP‐LC3

Cardiomyocytes cultured on confocal dishes were transduced with Ad‐GFP‐LC3 or Ad‐GFP‐mRFP‐LC3 28 hrs before treatment with or without Luteolin (8 μmol/l; Sigma‐Aldrich). After 48 hrs, cardiomyocytes were transferred into an ischaemic buffer and simulated ischaemia was performed in a humidified cell culture incubator (5% O_2_, 95% CO_2_, 37°C) for 8 hrs. Phalloidin conjugated with Rhodamine (Cytoskeleton) was used to stain actin filaments. The fluorescence of GFP‐LC3 or GFP‐mRFP‐LC3 was observed under the Olympus (Japan) FV1000 laser confocal microscope.

### Detection of aggresomes and P62

The ProteoStat Aggresome Detection Kit (Enzo Life Sciences, Lörrach, Germany) was used to detect aggregated proteins within the aggresome and aggresome‐like structures as described previously 23. All procedures were conducted according to the manufacturer's instructions. The aggresome and aggresome‐like structures was stained red by the ProteoStat aggresome red fluorescent molecular dye. P62 was probed with primary anti‐P62 antibody (ab91526; Abcam, Cambridge, UK) and secondary antibody labelled with Alexa Fluor 647 dye (Life Technologies, Camarillo, USA). Actin filaments were stained by Phalloidin conjugated with FITC (Cytoskeleton). The co‐localization of aggresomes and P62 was observed with a laser‐scanning confocal microscope (Olympus FV1000).

### Determination of cardiomyocyte apoptosis

Cell Death Detection kit (Roche, Penzberg, Germany) was used to detect apoptosis rate 8 hrs after hypoxia as described previously 24.

### Echocardiography

Four weeks after MI, mice were sedated with 3% isoflurane inhalation and studied on an echocardiography system (Sequoia Acuson, 15‐MHz linear transducer; Siemens, Erlangen, Germany). LV end‐systolic diameter (LVESD) and LV end‐systolic diameter (LVESD) were measured on the LV short axis. LV ejection fraction (LVEF) and LV fraction shortening (LVFS) were calculated by the use of computer algorithms.

### Western blot evaluation

Eight hours after hypoxia, protein was isolated from cardiomyocytes with standard Invitrogen protocols (Invitrogen, Carlsbad, CA, USA). Protein concentration quantitation was modified by Bradford assay (Bio‐Rad Laboratories, Hercules, CA, USA), protein was then separated by SDSPAGE with antibodies against Mst1 (ab51134; Abcam), p‐Mst1 (Thr183) (#110687; Sigma‐Aldrich), LC3A/B (#12741S, CST), P62 (ab91526; Abcam), Beclin1 (ab62472; Abcam), GAPDH (ab181602; Abcam), Caspase‐3(ab4051; Abcam), Cleaved caspase‐3 (ab2302; Abcam), Bax (ab5714; Abcam), Bcl‐2 (ab7973; Abcam). The blots were visualized with a chemiluminescene system (Amersham Bioscience, Buchinghamshire, UK). The signals were quantified by Image Pro Plus software (Media Cybernetics, MD Rockville, USA).

### Lactate dehydrogenase and CK‐MB release evaluation

Myocardium damage was assessed by lactate dehydrogenase (LDH) release and creatine kinase‐MB (CK‐MB) activities in plasma. Lactate dehydrogenase and CK‐MB activities were measured with commercial reagent kits which had adapted to spectrophotometric Auto analyzer (Sigma‐Aldrich) 3 hrs after MI.

### Determination of tissue myeloperoxidase, interleukin‐1α and tumour necrosis factor‐alpha activity

Tissue samples were taken from the indicated zones for myeloperoxidase (MPO) 3 hrs after MI. The activity of MPO was measured spectrophotometrically at 460 nm and expressed as units per 100 mg of tissue as described previously 13. The concentrations of interleukin‐1α (IL‐1α) and tumour necrosis factor‐alpha (TNF‐α) were assessed by ELISA according to the manufacturer's instructions 8. Values are expressed as pg/mg of total protein.

### *In vitro* citrate synthase, chain complex activities and ATP content

Citrate synthase (CS) and electron transport chain complex activities (Complex I, II, III, IV and V) were detected by using a CS activity assay kit (Sigma‐Aldrich). An adenosine triphosphate (ATP) bioluminescent assay kit (Beyotime, Shanghai, China) was used to measure the ATP content of the myocardium according to the standard protocols.

### Determination of mitochondrial transmembrane potential (ΔΨm)

Tetrechloro‐tetraethylbenzimidazol carbocyanine iodide (JC‐1) a cationic fluorescent dye, was used to detected the changes inΔΨm. Briefly, cardiomyocytes cultured on confocal dishes were subjected to hypoxia for 8 hrs after treatment with or without Luteolin (8 μmol/l; Sigma‐Aldrich). After hypoxia, the cells were incubated with JC‐1 and incubated for 20 min. at 37°C and observed under the Olympus FV1000 laser confocal microscope. The JC‐1 aggregates stained as red fluorescence represents highΔΨm, whereas green fluorescence represents JC‐I monomers in cells with lowΔΨm.

### Statistical analysis

Continuous variables were expressed as mean ± SD. Comparison between groups were subjected to anova followed by Bonferroni correction for post hoc *t*‐test. Data expressed as proportions were assessed with a chi‐squared test. Two‐sided tests have been used throughout, and *P* < 0.05 was considered statistically significant. SPSS software package version 14.0 (SPSS, Chicago, IL, USA) was used for data analysis.

## Results

### Treatment with Luteolin improves cardiac function in WT mice after MI

**Figure 1:**
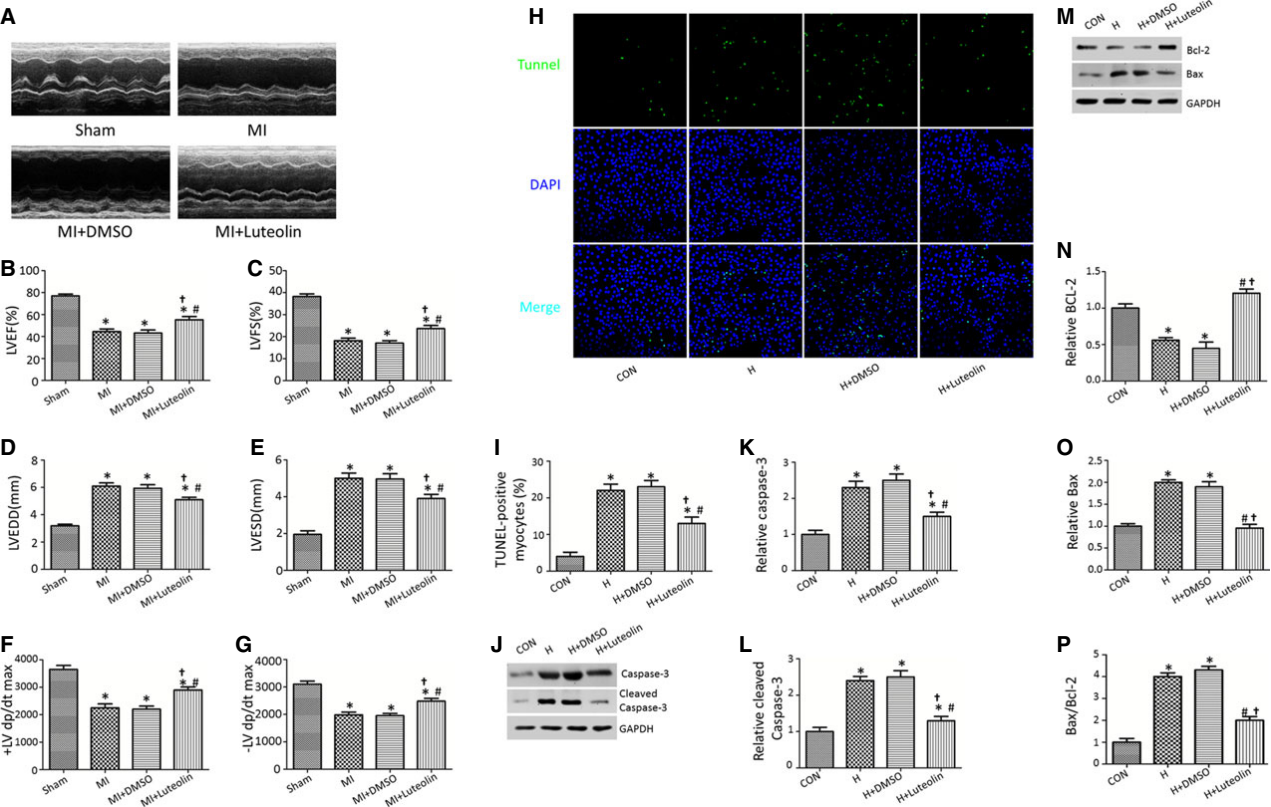
Luteolin improves cardiac function and mitigates left ventricle remodelling in mice after MI. (**A**) Representative echocardiographic images at 4 weeks after MI; (**B**) LV ejection fraction (LVEF); (**C**) LV fraction shortening (LVFS); (**D**) LV end‐diastolic diameter (LVEDD); (**E**) LV end‐systolic diameter (LVESD); (**F** and **G**) first derivative of the LV pressure (± LV dp/dt max); (**H**) Luteolin decreased TUNEL‐positive cardiomyocytes subjected to hypoxia; (**I**) Quantitative analysis of apoptotic index; (**J**–**P**) Protein expression with representative gel blots of Caspase‐3, Cleaved Caspase‐3, Bcl‐2, Bax and GAPDH; LV: left ventricular; H: Hypoxia. *P < 0.05 *versus* Sham group; ^#^ *P* < 0.05 *versus* MI (H) group. ^†^ *P* < 0.05 *versus* MI+DMSO (H+DMSO) group.

Four weeks of permanent coronary ligation caused severe cardiac dysfunction as evidenced by decreased LVEF, LVFS and ± LV dp/dt max with increased LVEDD and LVESD (Fig. 1A–G). Luteolin significantly increased LVEF, LVFS and ± LV dp/dt max, suggesting that treatment with Luteolin significantly improved LV systolic function after MI (Fig. 1A–C, F and G). In Luteolin‐treated mice, the increases of LVEDD and LVESD were significantly attenuated as compared with the vehicle‐treated mice (Fig. 1D and E), suggesting that Luteolin significantly attenuated LV remodelling after MI.

Creatine kinase‐MB, IL‐1α, LDH, MPO and TNF‐α were significantly reduced in the Luteolin‐treated group (Fig. S1A–E). Consistent with these observations, treatment with Luteolin significantly reduced cardiomyocytes apoptosis induced by hypoxia *in vitro*, as determined by TUNEL staining and caspase‐3 activity (Fig. 1H–L). The significantly decreased ratio of pro‐ to anti‐apoptotic protein (Bax/Bcl‐2), Bax protein level and increased Bcl‐2 protein level were also observed in the H+Luteolin group compared with the vehicle‐treated group (Fig. 1M–P).

### Luteolin administration attenuates cardiac dysfunction in the Mst1Tg mice without affecting the Mst1 Knockout (Mst1^−/−^) mice

**Figure 2:**
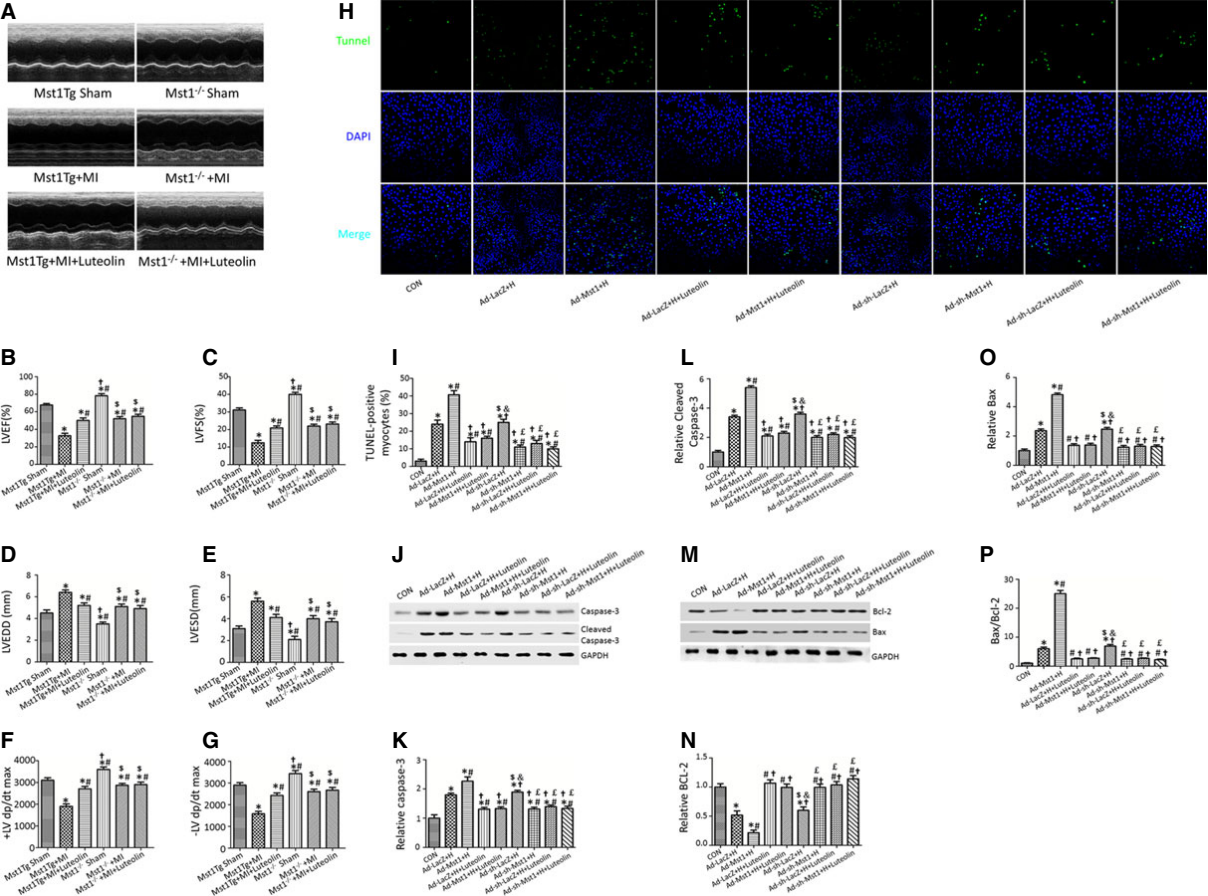
Luteolin attenuates adverse left ventricle remodelling and LV dysfunction through inhibiting Mst1 phosphorylation. (**A**–**G**) Luteolin promoted systolic function, attenuated LV dilation in the Mst1Tg mice, not in the Mst1^−/−^ mice 4 weeks after MI; (**H**) Luteolin decreased cardiomyocytes apoptosis index in Mst1 overexpression (Ad‐Mst1) group, but not in Mst1 knockdown group (Ad‐sh‐Mst1) after hypoxia; (**I**) Quantitative analysis of apoptotic index; (**J**–**P**) Quantitative analysis of protein expression with representative gel blots of Caspase‐3, Cleaved Caspase‐3, Bcl‐2, Bax and GAPDH; *P < 0.05 *versus* Mst1 Tg Sham (control) group; ^#^ *P* < 0.05 *versus* Mst1Tg + MI (Ad‐ LacZ + H) group; ^†^ *P* < 0.05 *versus* Mst1Tg + MI+Luteolin (Ad‐ Mst1+ H) group; ^$^ *P* < 0.05 *versus* Mst1^−/−^Sham (Ad‐LacZ + H +Luteolin) group; ^&^ *P* < 0.05 *versus* Ad‐Mst1+ H +Luteolin group; ^£^ *P* < 0.05 *versus* Ad‐sh‐LacZ + H group.

We next examined whether Luteolin improved cardiac function through Mst1 inhibition. After 4 weeks of coronary ligation, both LVEF and LVFS were significantly decreased in the Mst1Tg and the Mst1^−/−^ hearts subjected to MI injury (Fig. 2A–C). Specifically, Luteolin significantly increased the LVEF and LVFS in the Mst1Tg mice, slightly but insignificantly improved LVEF and LVFS in the Mst1^−/−^ mice (Fig. 2A–C). Increased LVEDD and LVESD in the Mst1Tg mice subjected to MI were blunted by Luteolin administration (Fig. 2D and E). As summarized in Figure 2, Luteolin markedly increased the ± LV dp/dt max in the Mst1Tg mice without affecting the Mst1^−/−^ mice underwent MI injury (Fig. 2F and G). The Mst1Tg mice shared the highest levels of the enzyme and cytokines including CK‐MB, IL‐1α, LDH, MPO and TNF‐α (Fig. S2A–E). Luteolin attenuated the up‐regulation of these cytokines solely in the Mst1Tg mice, suggesting that Luteolin inhibited the release of inflammatory cytokines in the remodelling heart through Mst1 suppression (Fig. S2A–E). Furthermore, Luteolin reduced cardiomyocytes apoptosis in the Ad‐Mst1‐treated cardiomyocytes whithout affecting the Ad‐sh‐Mst1‐treated cardiomyocytes (Fig. 2H and I). Coincidentally, Luteolin administration decreased the Bax/Bcl‐2 ratio, Cleaved Caspase‐3, Caspase‐3, Bax protein levels and increased Bcl‐2 protein level in the Ad‐Mst1‐treated cardiomyocytes whithout affecting the Ad‐sh‐Mst1‐treated cardiomyocytes (Fig. 2J–P).

### Luteolin stimulates autophagic flux in the cardiomyocytes subjected to hypoxia injury

**Figure 3:**
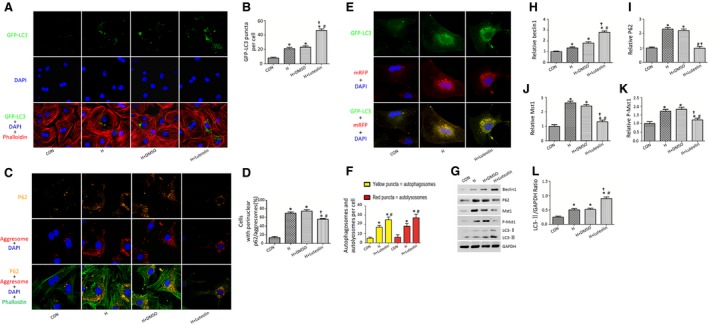
Luteolin enhances cardiomyocytes autophagy after hypoxia. (**A**) Luteolin increased the numbers of GFP‐LC3 puncta in cardiomyocytes transduced with Ad‐Mst1 after hypoxia; (**B**) Quantitative analysis of the number of GFP‐LC3 puncta; (**C**) Luteolin decreased the accumulation of P62 (orange) and aggresomes (red) in cardiomyocytes underwent hypoxia injury; (**D**) Quantitative analysis of the number of cells positively stained with P62 and aggresomes. (**E**) Luteolin increased the numbers of red and green puncta in cardiomyocytes transduced with Ad‐GFP‐mRFP ‐LC3 after hypoxia. (**F**) Quantitative analysis of the mean numbers of autophagosomes and autolysosomes. (**G**–**L**) Representative Immunoblots and quantitative analyses of Beclin1, P62, Mst1, p‐Mst1 and LC3‐II/GAPDH. *P < 0.05 *versus* control group; ^#^ *P* < 0.05 *versus* H group; ^†^ *P* < 0.05 *versus* H+DMSO group.

More GFP‐LC3 puncta and increased accumulation of aggresomes and P62 were observed in the hypoxia group as compared with the control group (Fig. 3A–D). Western blot analysis showed increased Beclin1, P62, Mst1, p‐Mst1 and LC3‐II protein levels in the hypoxia group as compared with the control group (Fig. 3G–L). Consistently, *in vivo* studies using Western blot analysis also demonstrated that MI increased Beclin1, P62, Mst1, p‐Mst1 and LC3‐II protein levels (data not shown). Interestingly, Luteolin pre‐treatment significantly increased the number of GFP‐LC3 puncta, attenuated the accumulation of aggresomes and P62 in the cardiomyocytes subjected to hypoxia injury (Fig. 3A–D). Luteolin pre‐treatment further increased the protein levels of LC3‐II and beclin1, whereas it decreased Mst1, p‐Mst1 and P62 protein levels (Fig. 3G–L), in the cardiomyocytes subjected to hypoxia injury (consistent *in vivo* data not shown).

In cardiomyocytes expressing GFP‐mRFP‐LC3, LC3 associated with autophagosomes can be visualized as puncta that are both red and green (appearing yellow in the merged image), whereas autolysosomes are visualized as puncta that are red only. In cardiomyocytes transduced with Ad‐GFP‐mRFP‐LC3, Luteolin pre‐treatment significantly increased the number of both green and red puncta as compared to control cardiomyocytes (Fig. 3E and F). The stimulated autophagic flux exerted by Luteolin administration was also demonstrated by increased LC3‐II expression and decreased P62 expression in the presence of bafilomycin A1, a lysosomal inhibitor used to evaluate autophagic flux (Fig. 3A–C).

### Luteolin enhances cardiomyocytes autophagy through Mst1 suppression

**Figure 4:**
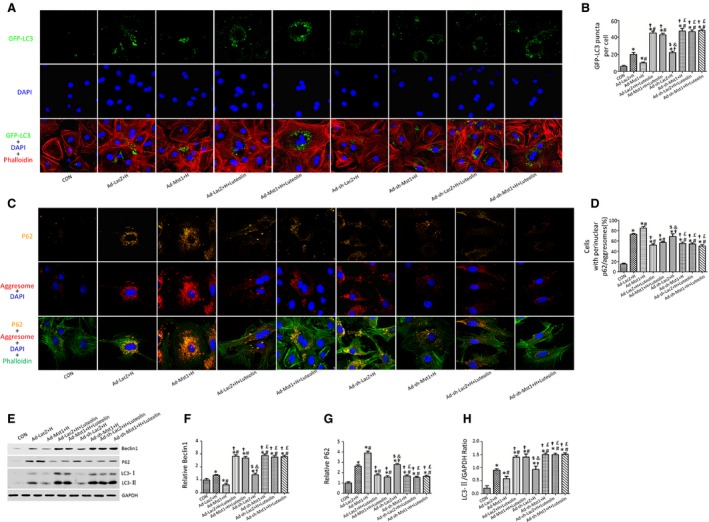
Luteolin augments cardiomyocytes autophagy subjected to hypoxia through Mst1 inhibition. (**A**) Luteolin increased the number of GFP‐LC3 puncta in the Ad‐Mst1 group; (**B**) Quantitative analysis of the number of GFP‐LC3 puncta; (**C**) Luteolin decreased the accumulation of P62 and aggresomes in the Ad‐Mst1 group; (**D**) Quantitative analysis of the number of cells positively stained with P62 and aggresomes; (**E**–**H**) Representative immunoblots and quantitative analyses of Beclin1, P62 and LC3‐II/GAPDH. *P < 0.05 *versus* control group; ^#^ *P* < 0.05 *versus* Ad‐ LacZ + H group; ^†^ *P* < 0.05 *versus* Ad‐ Mst1+ H group; ^$^ *P* < 0.05 *versus* Ad‐LacZ + H +Luteolin group; ^&^ *P* < 0.05 *versus* Ad‐Mst1+ H +Luteolin group; ^£^ *P* < 0.05 *versus* Ad‐sh‐LacZ + H group.

To investigate whether Luteolin enhanced autophagy through Mst1 inhibition, we transduced the neonatal rat cardiomyocytes with adenoviruses harbouring Mst1 (Ad‐Mst1), Mst1shRNA (Ad‐sh‐Mst1), LacZ (Ad‐LacZ) and LacZ shRNA (Ad‐sh‐LacZ). In cardiomyocytes transduced with GFP‐LC3, Mst1 overexpression significantly decreased the number of green puncta compared with the cardiomyocytes transduced with LacZ after hypoxia (Fig. 4A and B). Luteolin attenuated the suppression of autophagy induced by overexpression of Mst1, as demonstrated by more GFP‐LC3 puncta in the Ad‐Mst1+H+Luteolin group as compared to the Ad‐Mst1+H group (Fig. 4A and B). The Ad‐Mst1‐transduced cardiomyocytes showed the most prominent accumulation of aggresomes and P62 (Fig. 4C and D). Luteolin significantly reduced the accumulation of aggresomes and P62 in the cardiomyocytes transduced with adenoviruses harbouring Mst1 after hypoxia (Fig. 4C and D). However, Luteolin became completely ineffective in cardiomyocytes transduced with adenovirus harbouring Mst1shRNA (Fig. 4A and B). Moreover, Luteolin significantly increased the LC3‐II, Beclin1 protein levels and decreased P62 protein level in the Ad‐Mst1+H+Luteolin group without affecting the sh‐Mst1 transduced cardiomyocytes (Fig. 4E–H).

### Luteolin significantly improves mitochondria function of cardiomyocytes after hypoxia

**Figure 5:**
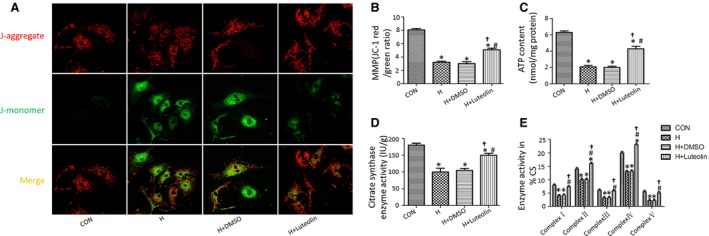
Luteolin improves mitochondrial function in cardiomyocytes after hypoxia. (**A**) Representative images of JC‐1 staining; (**B**) The ratio of aggregated and monomeric JC‐1; (**C** and **D**) ATP content and citrate synthase (CS) activity; (**E**) Enzymatic activities of complexes I–V in cardiomyocyte mitochondria. *P < 0.05 *versus* control group; ^#^ *P* < 0.05 *versus* H group; ^†^ *P* < 0.05 *versus* H+DMSO group.

Mitochondrial dysfunction plays a key role in cardiac remodelling after MI. As shown in Figure 5, the mitochondrial membrane potential was determined by JC‐1 staining. JC‐1 polymers representing intact mitochondrial membrane potential was stained as red fluorescence, whereas JC‐1 monomers indicating the dissipation of mitochondrial transmembrane potential was stained as green fluorescence. Luteolin significantly increased the ratio of JC‐1 red to JC‐1 green after hypoxia (Fig. 5A and B). The ATP content, CS activity and complexes I/II/III/IV/V activities of the cardiomyocyte mitochondria were significantly increased in the Luteolin‐treated group as compared to the cardiomyocytes treated with or without DMSO after hypoxia (Fig. 5C–E).

### Luteolin treatment attenuates mitochondria dysfunction through Mst1 inhibition

**Figure 6:**
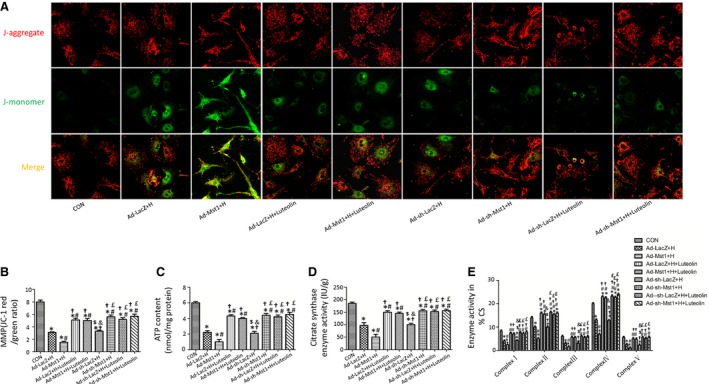
Luteolin promotes mitochondrial function in cardiomyocytes after hypoxia by inhibition of Mst1. (**A**) Luteolin improved mitochondrial membrane potential in Mst1 overexpression group; (**B**) the ratio of aggregated and monomeric JC‐1; (**C** and **D**) ATP content and citrate synthase (CS) activity; (**E**) Enzymatic activities of complexes I–V in cardiomyocyte mitochondria. *P < 0.05 *versus* control group; ^#^ *P* < 0.05 *versus* Ad‐ LacZ + H group; ^†^ *P* < 0.05 *versus* Ad‐ Mst1+ H group; ^$^ *P* < 0.05 *versus* Ad‐LacZ + H +Luteolin group; ^&^ *P* < 0.05 *versus* Ad‐Mst1+ H +Luteolin group; ^£^ *P* < 0.05 *versus* Ad‐sh‐LacZ + H group.

Mitochondrial dysfunction induced by hypoxia was demonstrated by lower ratio of JC‐1 red to JC‐1 green (Fig. 6A). Cardiomyocytes transduced with Ad‐Mst1 showed the lowest ratio of JC‐1 red to JC‐1 green after hypoxia. Luteolin significantly improved the mitochondrial membrane potential of the cardiomyocytes transduced with Ad‐Mst1 vector (Fig. 6A and B). Luteolin remarkably increased ATP content, CS activity and complexes I/II/III/IV/V activities in the cardiomyocytes transduced with adenoviruses harbouring Mst1 after hypoxia without affecting the cardiomyocytes transduced with adenovirus harbouring Mst1 shRNA (Fig. 6C–E).

## Discussion

Acute MI (AMI) is one of the major health care problems worldwide. It is fundamental to clarify the mechanisms regulating cardiomyocyte death and survival during ischaemic injury, thus to reduce the amount of myocardial loss after a sudden coronary occlusion 25. Cardiac dysfunction is viewed as a well‐recognized and common complication following AMI which is associated with substantially higher fatality rate 2, 26, 27, 28. Adverse remodelling of the left ventricle was characterized by chamber dilation and LV dysfunction after MI 29. In the present study, pre‐treatment with Luteolin improved cardiac function as demonstrated by echocardiography and haemodynamic measurements. The myocardial inflammatory cytokines release was decreased in the Luteolin‐administrated mice subjected to MI injury. These results indicated that Luteolin mitigated cardiac dysfunction and adverse remodelling after MI.

Autophagy is a degradation mechanism of lysosome‐dependent turnover of damaged protein aggregates and organelles, which is critical to cell survival by maintaining organelle function and protein quality 30. Suppression of autophagy below physiological levels leads to heart failure, suggesting that autophagy exerts a patent role in maintaining cardiac function 30.

Up‐regulation of autophagy in response to various pathological conditions including ischaemic injury and heart failure is generally compensatory, thus reducing energy loss and eliminating damaged organelles and protein aggregates 17, 31. Luteolin has been proven to modulate autophagy in many other circumstances. Luteolin protected mice brain from traumatic brain injury by up‐regulating autophagy 20. Luteolin also stimulated the autophagic process in the metastatic MET4 cells 32. Consistently, the present study also demonstrated that MI induced up‐regulation of cardiomyocyte autophagy which was indicated by increased LC3‐II and beclin1 protein level. Cardiomyocytes damage induced by hypoxia is often accompanied by accumulation of aggresomes and P62 which indicated insufficient autophagy. Interestingly, Luteolin pre‐treatment significantly promoted neonatal cardiomyocyte autophagy after hypoxia, as demonstrated by more GFP‐LC3 puncta, less accumulation of aggresomes and P62. Western blot analysis also demonstrated that Luteolin up‐regulated autophagy in the cardiomyocytes subjected to simulated MI injury. Furthermore, Luteolin increased autophagosomes formation in Ad‐GFP‐mRFP‐LC3‐transduced cardiomyocytes subjected to simulated MI injury. Luteolin stimulated autophagic flux in the presence of bafilomycin A1, which is a lysosomal inhibitor used to evaluate autophagic flux.

Mammalian sterile 20‐like kinase 1 is a component of the Hippo signalling pathway, which promotes cardiac dysfunction by inhibiting autophagy 23. We then constructed MI injury model in the Mst1Tg and Mst1^−/−^ mice with or without Luteolin pre‐treatment to elucidate the molecular mechanism underlying the protective effects of Luteolin. We employed immunofluorescence and Western blot analysis to determine cardiomyocyte autophagy among different groups. Autophagy was found to be enhanced after Luteolin treatment in the Mst1Tg mice underwent MI injury. However, in the Mst1^−/−^ mice, Luteolin failed to up‐regulate cardiomyocyte autophagy. These results indicated that Luteolin may exert its protective effects against MI through Mst1 inhibition.

In line with these observations, Luteolin improved cardiac function, decreased LDH release, reduced inflammatory cytokines release solely in the Mst1Tg mice subjected to MI injury. These results further implied that Luteolin protected cardiomyocyte from MI injury by inhibiting Mst1 phosphorylation. Luteolin‐induced autophagy might play a pivotal role in its protection effects.

Mitochondria are the most abundant organelle in the heart and around ninety per cent of ATP were produced by oxidative phosphorylation in the mitochondria 33. Mitochondria are involved in a wide spectrum of cellular functions and thus play a key role in mediating cellular homoeostasis. Mitochondria have been demonstrated to regulate autophagy, apoptosis and intracellular signalling in the cardiomyocytes 34, 35, 36. As a result, maintenance of mitochondria biogenesis and function are essential for cardiomyocytes survival. In this study, Luteolin increased mitochondrial membrane potential, ATP content, CS activity and complexes I/II/III/IV/V activities in the cardiomyocytes subjected to simulated MI injury. Interestingly, the protective effects of Luteolin on mitochondrial membrane potential and biogenesis were abolished by Mst1 knockout. Substantial loss of cardiac myocytes after MI can lead to contractile dysfunction and heart failure. Mst1 is one of the protein kinases most strongly activated during cardiomyocyte apoptosis 18, 19, 37. Our previous study also proved the anti‐apoptotic effects of Luteolin by activating PI3K/Akt pathway while increasing Bcl‐2‐associated death promoter (BAD) phosphorylation and decreasing the ratio of Bax to Bcl‐2 13. In agreement with our previous results, Luteolin inhibited cardiomyocytes apoptosis. Mst1 knockout abolished the anti‐apoptotic effects of Luteolin. Thus, Luteolin may also exert anti‐apoptotic effects by Mst1 inhibition.

In conclusion, this study provides the evidence that Luteolin enhances cardiac function, reduces cardiac enzyme and inflammatory markers release after MI. The protective effects of Luteolin are associated with up‐regulation of autophagy, down‐regulation of apoptosis and improvement of mitochondrial biogenesis through Mst1 inhibition. Luteolin may represent a potential drug to treat or prevent development of cardiac dysfunction and adverse cardiac remodelling caused by MI.

## Conflicts of interest

None declared.

## Supporting information


**Figure S1** Luteolin reduces the release of cardiac enzyme and inflammatory cytokines after myocardial infarction (MI).
**Figure S2** Luteolin reduces the release of cardiac enzyme and inflammatory cytokines through Mst1 inhibition.
**Figure S3** Luteolin simulates autophagic flux in cardiomyocytes after hypoxia.
