# Supplementary material for: Luteolin alleviates post‐infarction cardiac dysfunction by up‐regulating autophagy through Mst1 inhibition
Source: J Cell Mol Med. 2015 Nov 5;20(1):147–56. doi: 10.1111/jcmm.12714 (PMC4717847; doi:10.1111/jcmm.12714)
Supplement: Supplementary file 1 — Figure S1 Luteolin reduces the release of cardiac enzyme and inflammatory cytokines after myocardial infarction (MI). Figure S2 Luteolin reduces the release of cardiac enzyme and inflammatory cytokines through Mst1 inhibition. Figure S3 Luteolin simulates autophagic flux in cardiomyocytes after hypoxia. [file JCMM-20-147-s001.docx]

**Supplemental Materials**


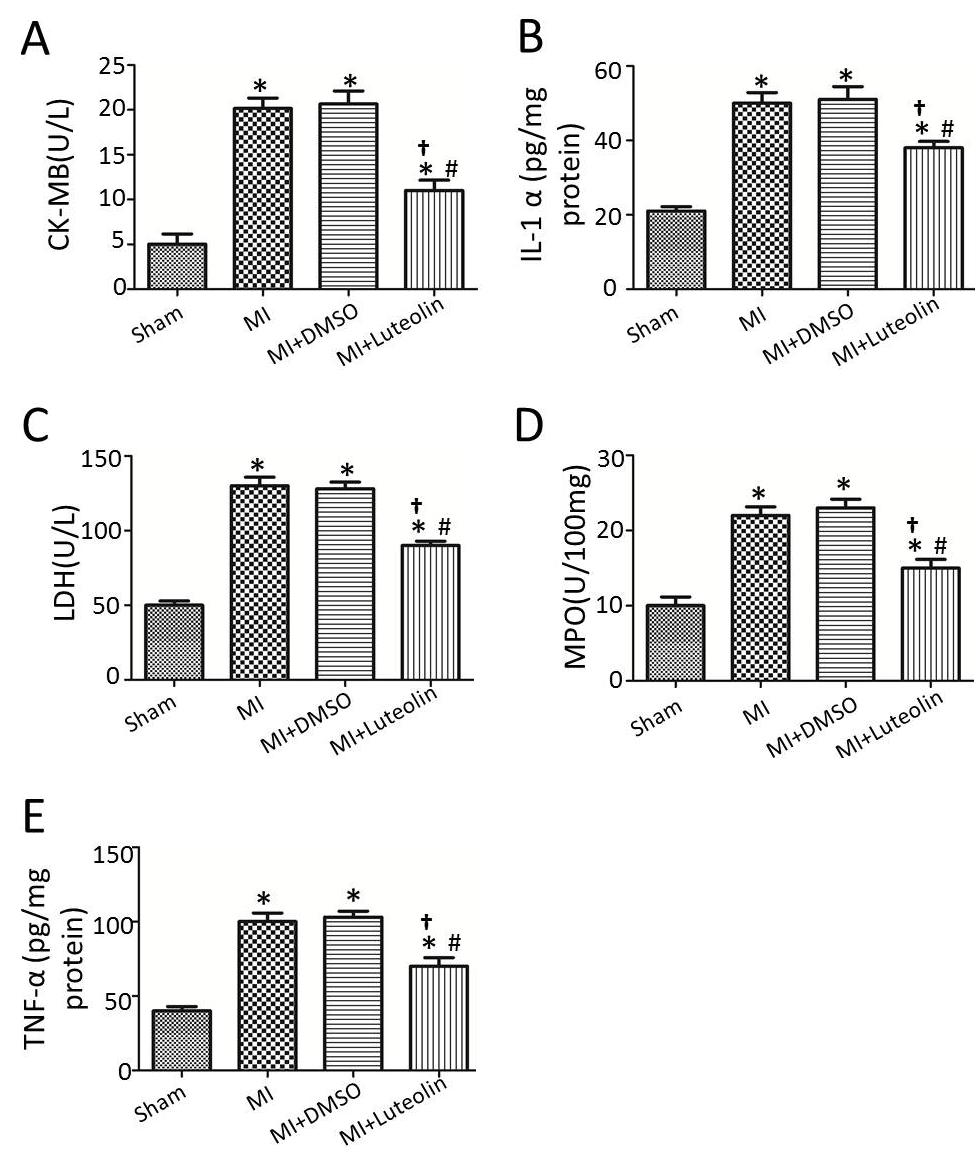


**Supplementary Figure 1 Luteolin reduces the release of cardiac enzyme and inflammatory cytokines after myocardial infarction (MI)**

A, CK-MB; B, interleukin (IL)-1α; C, LDH; D, MPO; E, TNF-α. *P<0.05 vs control group; #P<0.05 vs MI group; †P<0.05 vs MI+DMSO group.


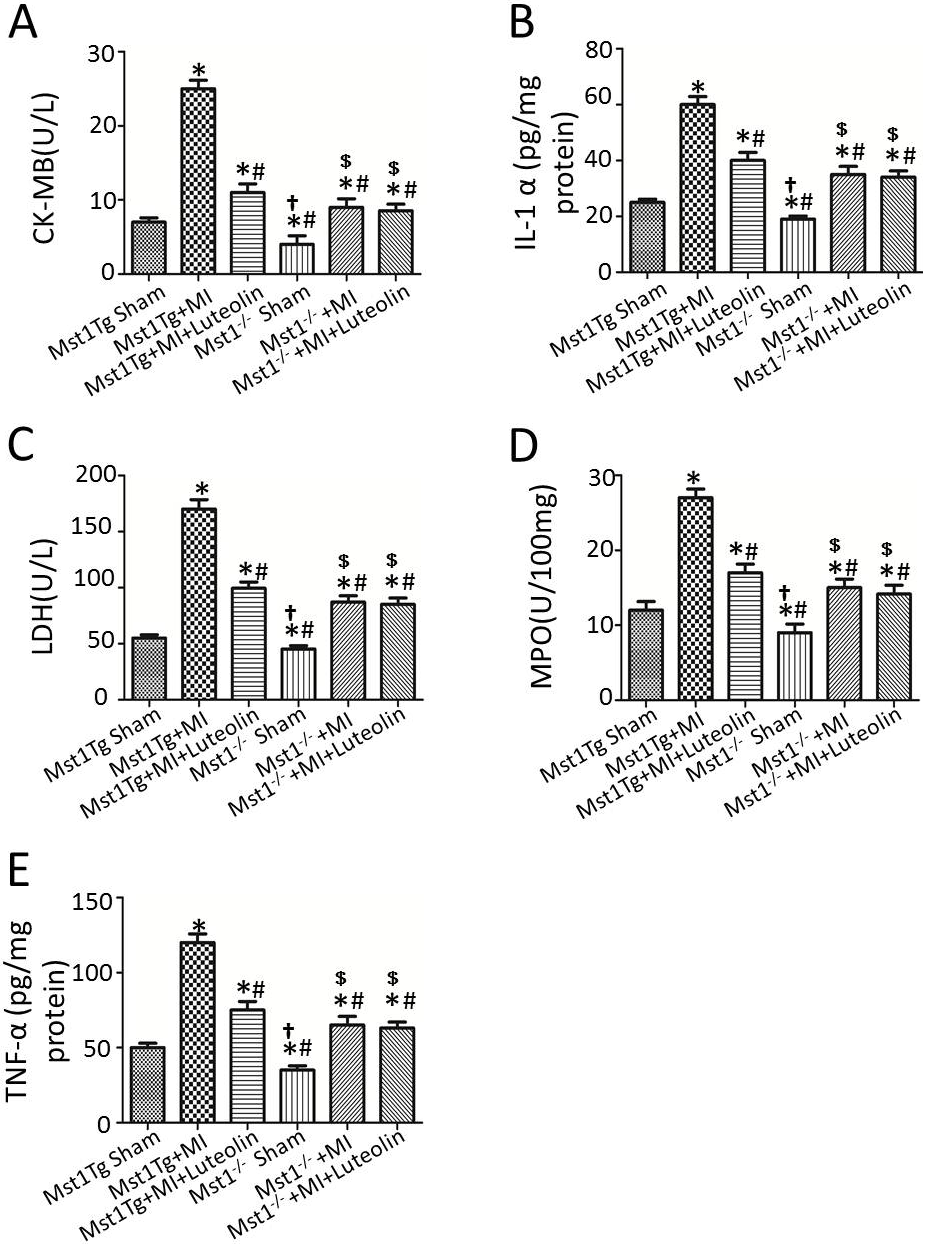


**Supplementary Figure 2 Luteolin reduces the release of cardiac enzyme and inflammatory cytokines through Mst1 inhibition**

A, CK-MB; B, interleukin (IL)-1α; C, LDH; D, MPO; E, TNF-α. *P<0.05 vs Mst1Tg Sham group; #P<0.05 vs Mst1Tg + MI group; †P<0.05 vs Mst1Tg + MI + Luteolin group; ﹩P<0.05 vs Mst1^-/-^ Sham group.


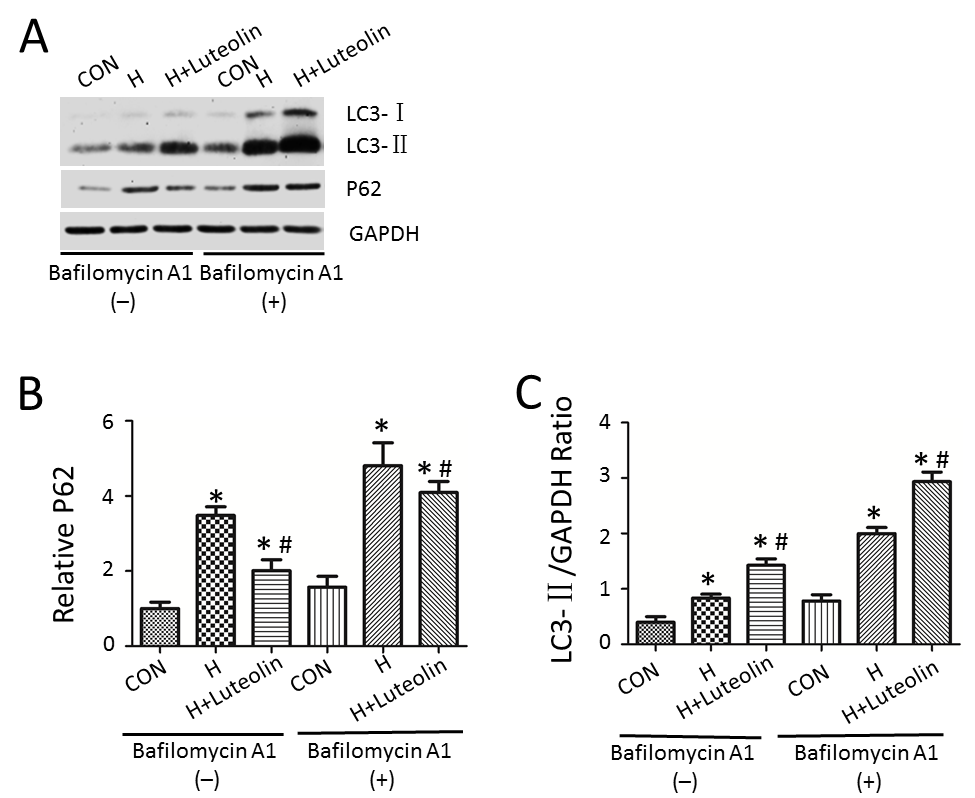


**Supplementary Figure 3 Luteolin simulates autophagic flux in cardiomyocytes after hypoxia**

A-C, Representative Immunoblots and quantitative analyses of P62 and LC3-II/GAPDH in cardiomyocytes treated with or without bafilomycin A1. CON: control; H: hypoxia. *P<0.05 vs CON group; #P<0.05 vs H group.
